# MOLE 2.0: advanced approach for analysis of biomacromolecular channels

**DOI:** 10.1186/1758-2946-5-39

**Published:** 2013-08-16

**Authors:** David Sehnal, Radka Svobodová Vařeková, Karel Berka, Lukáš Pravda, Veronika Navrátilová, Pavel Banáš, Crina-Maria Ionescu, Michal Otyepka, Jaroslav Koča

**Affiliations:** 1 National Centre for Biomolecular Research, Faculty of Science and CEITEC-Central European Institute of Technology, Masaryk University Brno, Kamenice 5, 625 00 Brno-Bohunice, Czech Republic; 2 Faculty of Informatics, Masaryk University Brno, Botanická 68a, 602 00 Brno, Czech Republic; 3 Department of Physical Chemistry, Regional Centre of Advanced Technologies and Materials, Faculty of Science, Palacký University Olomouc, tř. 17. listopadu 12, 771 46 Olomouc, Czech Republic

**Keywords:** Channels, Tunnels, Pores, Protein structures, Cytochrome P450, CAM, BM3

## Abstract

**Background:**

Channels and pores in biomacromolecules (proteins, nucleic acids and their complexes) play significant biological roles, e.g., in molecular recognition and enzyme substrate specificity.

**Results:**

We present an advanced software tool entitled MOLE 2.0, which has been designed to analyze molecular channels and pores. Benchmark tests against other available software tools showed that MOLE 2.0 is by comparison quicker, more robust and more versatile. As a new feature, MOLE 2.0 estimates physicochemical properties of the identified channels, i.e., hydropathy, hydrophobicity, polarity, charge, and mutability. We also assessed the variability in physicochemical properties of eighty X-ray structures of two members of the cytochrome P450 superfamily.

**Conclusion:**

Estimated physicochemical properties of the identified channels in the selected biomacromolecules corresponded well with the known functions of the respective channels. Thus, the predicted physicochemical properties may provide useful information about the potential functions of identified channels. The MOLE 2.0 software is available at http://mole.chemi.muni.cz.

## Background

The number of known three-dimensional (3D) structures of biomacromolecules (proteins, nucleic acids and their complexes) has increased rapidly over recent years, enabling relationships between structure and function to be analyzed at an atomic level. The functions of biomacromolecules usually depend on interactions with other biomacromolecules as well as ions and small molecules, such as water, messenger and endogenous compounds, pollutants and drugs, which can occupy “otherwise empty spaces” in biomacromolecular structures [1]. Thus, information about the nature of empty spaces in a biomacromolecule can provide valuable insights into its functions.

**Figure 1:**
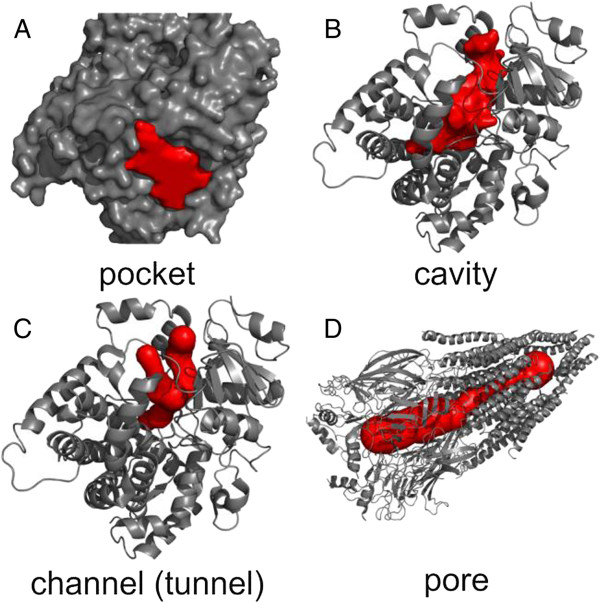
**Classification of biomacromolecular “empty spaces”: A) pockets, B) cavities, C) channels (or tunnels), and D) pores.**

Biomacromolecular empty spaces can be classified as pockets, cavities, voids, channels (tunnels) or pores (Figure 1). A *pocket* usually refers to a shallow depression on a biomacromolecular surface, whereas a *cavity* describes a deeper pocket or cleft. If the cavity is encapsulated inside a biomolecule (having no connection to a water accessible surface), it is called a *void*. A *channel* or *tunnel* is a pathway inside a cavity connecting an internal point (typically the deepest apex) with an exterior. A *pore* is considered here as a channel that passes through the biomacromolecule from one point on the surface to another.

The present work focused on pores and channels because they have been shown to play significant roles in many biologically relevant systems. For example, internal pores of ion channels maintain a highly selective ionic balance between the cell interior and exterior, [2-6] photosystem II channels are involved in photosynthesis, [7,8] ribosomal polypeptide exit channels allow nascent peptides to leave the ribosome during translation, [9] and active site access/egress channels enable substrate/product to enter/leave the occluded active sites of various enzymes (e.g., cytochrome P450, [10-15] acetylcholinesterase, [16-18] etc.). Information about the nature of active site access paths can also be utilized in biotechnology applications aimed at designing more effective and selective enzymes [19-21]. Unquestionably, identification and characterization of channels are fundamental to understanding numerous biologically relevant processes and serve as a starting point for rational drug design, protein engineering and biotechnological applications.

Over the last few years, numerous computational approaches have been developed for detection and characterization of empty spaces in biomacromolecules, particularly proteins [22]. The main strategies used in the developed algorithms can be grouped into four classes [23]. The first class comprises grid-based methods, which project biomacromolecular structures onto a 3D grid, process the void grid voxels and connect them into pockets or tunnels. These methods are used in numerous software tools, such as POCKET [24], LIGSITE [25,26], dxTuber [27], HOLLOW [28], 3V [29], CAVER 1.x [30] and CHUNNEL [31]. Sphere-filling methods belong to a second class. These methods carpet biomacromolecules with spheres layer by layer. A cluster of carpeting spheres is considered a pocket. This method is implemented in PASS [32] and SURFNET [33]. The third class involves slice and optimization methods. These methods split a biomacromolecular structure into slices along a start vector defined by the user and then optimization methods are used to determine the largest sphere. These approaches are implemented in the software HOLE [34] and PoreWalker [35]. The fourth class represents methods utilizing Voronoi diagrams, in which the shortest path is searched from a starting point to the biomacromolecular surface. This approach was used in the previous version of MOLE 1.x [19] and it is also utilized in other software tools, e.g., MolAxis [36,37], CAVER 2.0 [38] and CAVER 3.0 [39].

Here, we present an advanced and fully automatic software tool, MOLE 2.0, based on a new, fast and robust algorithm for finding channels and pores. MOLE 2.0 provides an improved approach for channel identification. The algorithm introduces several preprocessing steps that result in increased speed (up to several times faster), accuracy (more relevant channels are identified) and robustness. New capabilities include the computation of pores and better identification of channel start points. It contains extended options for starting point selection and allows improved computation of channel profiles together with estimation of their basic physicochemical properties. The implemented automatic filtering of obtained channels facilitates selection of the relevant channels. MOLE 2.0 offers an innovative user experience, as it can be used effectively even without knowledge of the underlying algorithms whilst at the same time allows the tunnel detection algorithm to be tweaked interactively, such that the results are immediately available for inspection and comparison. MOLE 2.0 also introduces a new, intuitive and user-friendly interface. MOLE 2.0 can be used as a stand-alone application or as a plugin for the widely used software PyMOL [40]. Some functionality is also available in a platform-independent manner via the web-based application MOLE*online* 2.0 [41].

## Implementation

### MOLE 2.0 algorithm

**Figure 2:**
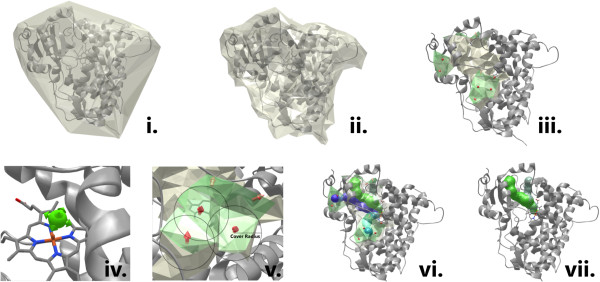
**Scheme showing the steps i-vii involved in the channel calculation algorithm (see the text for details).** Illustrated for cytochrome P450 3A4 (PDB ID: 1TQN).

The algorithm for finding channels implemented in MOLE 2.0 involves seven steps: i) computation of the Delaunay triangulation/Voronoi diagram of the atomic centers, ii) construction of the molecular surface, iii) identification of cavities, iv) identification of possible channel start points, v) identification of possible channel end points, vi) localization of channels, and vii) filtering of the localized channels (Figure 2).

#### Step i: computing the delaunay triangulation/voronoi diagram

In the first step, the Delaunay triangulation of the atomic centers is computed using an incremental algorithm that utilizes pre-sorted input points according to the Hilbert curve [19,42]. The Voronoi diagram is then constructed as the dual of the Delaunay triangulation. The Voronoi diagram can be represented as a graph with vertices corresponding to the circumcenters of the Delaunay tetrahedrons and edges present if two tetrahedrons share a common side (i.e., share exactly three vertices).

#### Steps ii and iii: approximating the molecular surface and identifying cavities

The molecular surface is approximated by iterative removal of boundary tetrahedrons from the outermost layers (i.e., tetrahedrons found at the interface between the molecule and the external environment). Boundary tetrahedrons produced by the triangulation are removed in this step if they are sufficiently large to fit a sphere with a given *probe radius* (tetrahedron *T* fits a sphere *S* with probe radius *r* if the center *C* of sphere *S* can be placed inside the tetrahedron and the distance to all vertices of *T* is greater than or equal to the sum of *r*, with the van der Waals radius of an atom corresponding to the given vertex). Next, tetrahedrons that are too small to fit a sphere with *interior radius* are removed. Remaining tetrahedrons form one or more connected components. We call the components that contain at least one tetrahedron on the molecular surface *cavity diagrams*. It should be noted here that the *cavity diagram* is a purely geometrical concept to help identify regions of space (volume) that can contain tunnels and only very loosely corresponds to the cavities shown in Figure 1B).

#### Steps iv and v: identifying possible start and end points of channels

The algorithm includes two ways to specify potential channel start and end points:

•*Computed*: Start and end points are defined as the centers of the *deepest* tetrahedrons in each cavity. The depth of the tetrahedron is defined as the number of Voronoi edges from the closest boundary tetrahedron.

•*User-defined*: Specified by a 3D point (that can also be defined as a centroid of several residues). Next, cavities that have at least one tetrahedron with a centroid within the *origin radius* from the user-specified point are found. Finally, for each such cavity, the start point is selected as the circumsphere center of the tetrahedron closest to the original point. Potential channel end points are placed in the centers of certain boundary tetrahedrons in such a way that the distance between two end points is at least the *cover radius*. This is achieved by picking the largest boundary tetrahedron and marking it as an exit, then removing all boundary tetrahedrons within the *cover radius*. This process is repeated until all non-exit boundary tetrahedrons are removed.

#### Step vi: computing channels

Once the potential start and end points have been identified, channels are computed as the shortest paths between all pairs of start and end points in the same cavity diagram. To achieve this, Dijkstra’s algorithm is used with edge weights given by the following formula:

(1) $$w\left(e\right)=\frac{l\left(e\right)}{d{\left(e\right)}^{2}+\epsilon },$$

where *l(e)* is the length of the edge, *d(e)* is the distance of the edge to the closest atom van der Waals sphere and *ϵ* is a small number to avoid division by zero [19].

At this stage, each channel is represented by a sequence of tetrahedrons. The next step is to approximate the channel centerline by a natural cubic spline of the circumsphere centers of the tetrahedrons. Additionally, a “radius spline” is computed that determines the centerline distance to the closest atom van der Waals sphere.

#### Step vii: filtering of channels

The above-described steps usually generate a large number of channels. However, many of these channels are either too narrow (i.e., have a bottleneck with a small radius) to be considered relevant or are duplicated (i.e., too similar to each other). To obtain the most relevant channels, the algorithm contains a filter with two criteria.

The first criterion deals with bottlenecks using parameters that define the maximum *bottleneck length* and minimum *bottleneck radius*. These two parameters ensure that there is enough room for a ligand to pass through each region of the tunnel.

The second criterion is necessary because channels generated using steps (i-vi) of the algorithm often have very similar centerlines that only deviate towards the ends of the channels near the molecular surface. Therefore, for practical purposes, these channels can be considered identical. To remove duplicate channels, a parameter called the *cutoff ratio* is introduced. The centerlines of each pair of tunnels are compared, and if two channels “share” at least the *cutoff ratio* percentage of the centerline, the longer one is removed.

### Lining and physicochemical properties of identified channels

The channel lining amino acids residues are the residues that surround the centerline of the channel. The centerline is uniformly divided into layers, and each layer is defined by the residues lining it. A new layer starts whenever there is a change in the list of residues lining the tunnel along its length. The lining of the channel is then described as a sequence of layer lining residues. For each layer, the length (distance between the first and last atom of the layer projected to the tunnel centerline) and radius (bottleneck) are computed. Additionally, the orientation of each residue is determined to check whether the residue faces the tunnel with its backbone or side-chain moiety.

Basic physicochemical properties of protein channels are computed from the set of lining amino acids residues. In MOLE 2.0, the *charge* according to the amino acid side-chain type (Arg, Lys +1*e*; Glu, Asp −1*e*), *hydropathy*[43], *hydrophobicity*[44], *mutability*[45] and *polarity*[46] are computed. The properties are calculated for the unique residues surrounding the channel by averaging tabulated values (Additional file 1: Table S1) for every amino acid residue that has a side chain oriented towards the tunnel. The only exception is charge, which is calculated as the sum of the charges of individual amino acid side chains. For amino acids that have their main chains oriented towards the tunnel, tabulated values for glycine (Gly) are used to compute the hydrophobicity and hydropathy, and the value for asparagine (Asn) is used to evaluate polarity. Amino acids residues that have their main chains lining the channel are not considered when computing mutability. MOLE 2.0 also enables calculation of the weighted physicochemical properties (except the charge) of the channel. The weighted properties are evaluated by applying the above methods separately for each layer and then computing the weighted average, where the weight is given by the length of the layer. We note that the calculated physicochemical properties should be interpreted with care, because the used calculation comes from an assumption that the side chains making the channel wall determine the internal environment of the channel.

### Merging channels to pores

The MOLE 2.0 algorithm can compute pores by merging channels. There are three modes for computing pores. The first automatic mode evaluates pores as “channels” between all pairs of end points in a given cavity. In the second mode channels are computed among a set of user-selected end points. Finally, the third mode first computes channels from a user-defined start point and then merges them to form a pore. This mode also imposes a so called “pore criterion” that stipulates that the end points of the pore must be further away than the average length of the channels that formed the pore. In all modes, pores that are too similar are removed using the same criteria as for channels.

### Complexity of the algorithm

The worst-case complexity of the algorithm is (*N*^2^ log *N*), where *N* is the number of atoms in the molecule. However, in most practical cases, the complexity is *O*(*M* log *M*), where *M* is the number of vertices in the Voronoi diagram. In the worst case, *M* = *N*^2^. However, as shown by Dwyer *et al.*[47], in most cases *M* = *O*(*N*). Thus, as a result of the use of the incremental algorithm and Hilbert curve ordering, the complexity of calculating the Delaunay triangulation of most molecular structures is *O*(*N* log *N*). Finally, the complexity of all the remaining steps of the algorithm is at most *O*(*M* log *M*).

**Figure 3:**
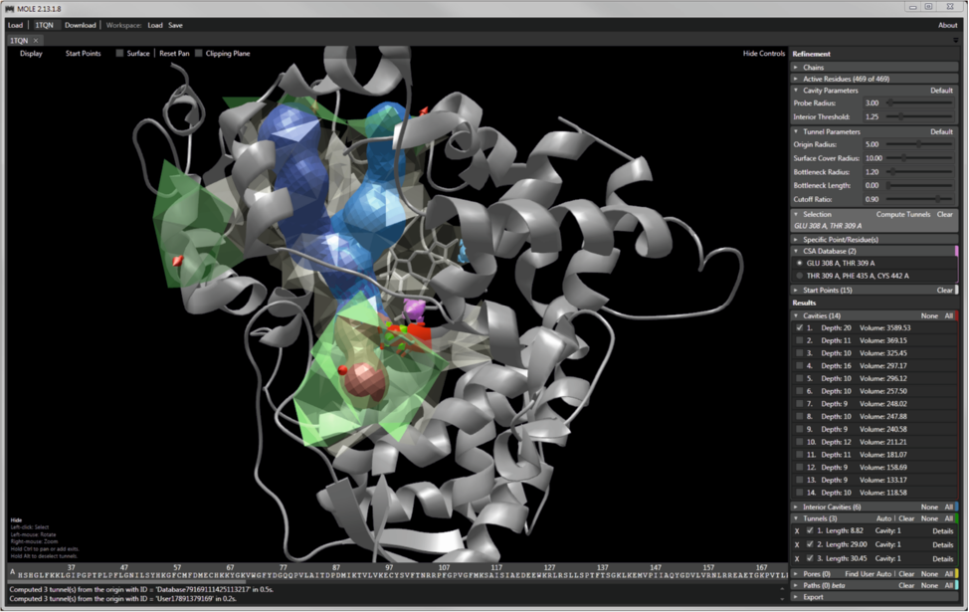
**MOLE 2.0 graphical user interface.** The left side of the window contains an interactive visualization of the molecule, cavities and computed tunnels. The panel on the right allows the user to tune the computation parameters, select which results are visualized and export them.

**Figure 4:**
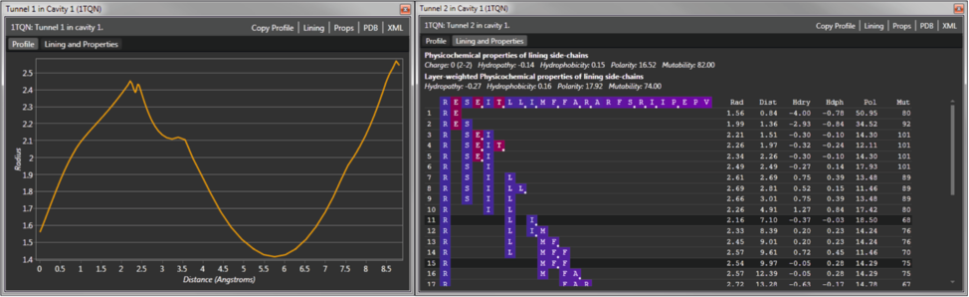
**MOLE 2.0 channel details.** Channel profile, i.e., plot of radius vs. distance from the start point (left), together with a list of lining amino acid residues and physicochemical properties (right).

MOLE 2.0 (Figure 3) supports protein files in PDB format. Once the protein is loaded, the GUI provides a full interactive 3D rendering of the protein and the option to tune individual parameters of the channel computation. The GUI displays information about the identified cavities and once channels or pores are computed, a detailed view of them can be displayed that provides information about the channel’s profile, lining and physicochemical properties (Figure 4). Information on the channels can be exported in several formats, including XML, CSV, PDB and PyMOL for enhanced visualization.

The command line version of MOLE 2.0 requires the user to specify the input parameters in an XML file. The output can be obtained in XML format as well as a PDB or PyMOL script together with 3D representations of channels that can be loaded to Jmol [48] (http://www.jmol.org). The complete documentation can be found on the web page http://mole.chemi.muni.cz.

### Case study: properties of channels of cytochrome P450s BM3 and P450cam

Channels were calculated using MOLE 2.0 with parameters set as follows: minimal bottleneck radius 1.25 Å, probe radius 3 Å, surface cover radius 10 Å and origin radius 5 Å. The heme cofactor was used as the start point in all structures, while all other non-protein (“HETATM”) groups were ignored. The PDB database contains a relatively large number of X-ray structures of the two selected cytochrome P450s: 43 structures with 54 chains for P450cam (CAM) and 37 structures with 80 chains for P450BM3 (BM3). All crystal structures were divided into monomers and superimposed using the PyMOL 0.99rc program [40]. The identified channels were sorted into specific families according to the nomenclature of Wade and coworkers [15]: channels were included in a particular family if they had at least one point that trespassed a 4 Å wide cube in space assigned to a specific area for that channel family (i.e., through the B/C loop for channel 2e). Only the shortest channel in each channel family was selected for each protein structure. Other similar channels were designated as duplicates. The remaining channels were visually checked and meandering channels were also removed. Duplicates were also excluded from the comparison of physicochemical properties.

## Results and discussion

### Benchmarking study

MOLE 2.0 was compared with four other software tools: MOLE 1.4 [19], MolAxis [36], CAVER 2.0 [38] and CAVER 3.0 [39] (beta version). The main features of the software tools are listed in Table 1. By comparison, MOLE 2.0 provides the richest set of input and output features and has the advantage that both command line and graphical user interfaces are available. The need for a start point is made easier by the fact that MOLE 2.0 enables active sites annotated in the Catalytic Site Atlas (CSA, http://www.ebi.ac.uk/thornton-srv/databases/CSA/) [49] to be used as well as automatic identification of start points in a given structure. Data generated by MOLE 2.0 can be exported to PyMOL [40], which is a popular visualization software, and conveniently, MOLE 2.0 can also be called directly from PyMOL via a plug-in module. In the MOLE 2.0 GUI, a user can select and change the channel end points, which may facilitate the detection of complex channels and pores. The calculation of channels can be customized through nine parameters, whose default values enable automatic identification of channels in many common protein structures. Hence, MOLE 2.0 can be readily used by a new user but provides sufficient flexibility for an advanced user. Besides setup of these parameters, users can adjust the surface of a molecule and filtering of detected channels. It should be noted that MOLE 2.0 is the only software currently available that allows a user to compute cavities and estimate physicochemical properties of identified channels.

**Table 1:** Basic features of software tools for channel identification

**Features**		**Software**				
		**MOLE 2.0**	**MOLE 1.4**	**MolAxis**	**CAVER 2.0**	**CAVER 3.0**
Input and output	Command line interface	Yes	Yes	Yes	Yes	Yes
	GUI	Yes	Web	Web	No	No
	Suggested start points from CSA	Yes	Yes	No	No	No
	Automatic suggestion of start points	Yes	No	Yes	No	No
	Possibility to set end point	Yes	No	No	No	No
	PyMOL export	Yes	Yes	No	Yes	Yes
	PyMOL plugin	Yes	Yes	No	Yes	Yes
Settings of calculation	Number of parameters	9	9	11	8	35
	Adjustable surface of a molecule	Yes	No	Limited	No	Yes
	Channel filtering	Yes	No	Limited	No	Yes
	Cavity computation	Yes	No	No	No	No
	Computation of physicochemical properties	Yes	No	No	No	No

**Figure 5:**
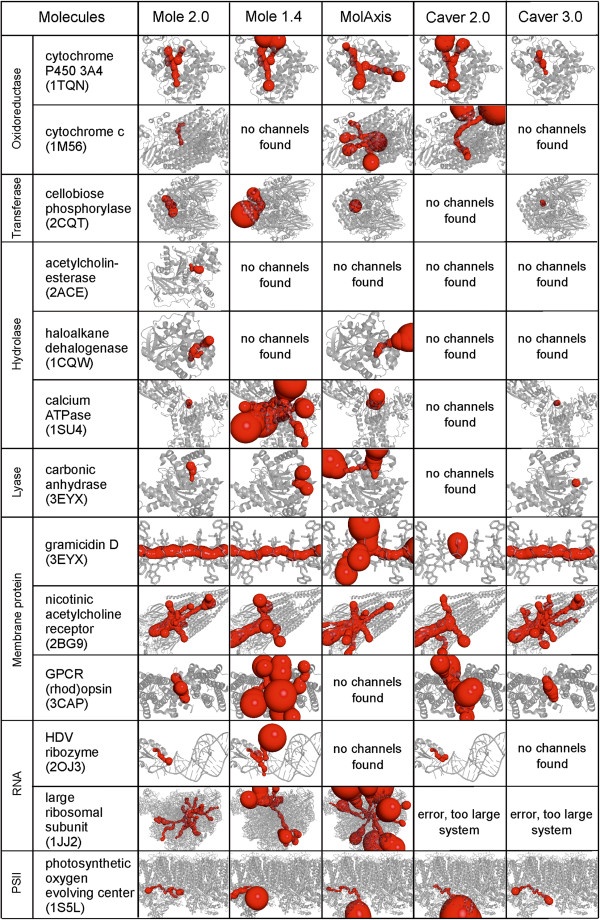
Channels found in the analyzed molecules.

The performance of all the considered software tools was compared on a set of thirteen diverse biomacromolecules containing several channels or pores: two RNAs, three membrane proteins, the photosystem II oxygen evolving center and seven representatives of enzymatic groups, which have all been targeted in research studies dealing with molecular channels (Figure 5). This comparison was carried out on the laptop with CPU Intel Core i5-430 M 2.26 GHz and 4GB RAM, running native Windows 7. For MolAxis, the webserver (http://bioinfo3d.cs.tau.ac.il/MolAxis/) was used. The software tools were used to identify channels with a radius of at least 1.25 Å along most of their length. Because some channels may be “partially closed” by an amino acid side chain, we also considered channels with a radius less than 1.25 Å provided this narrowing was not longer than 3 Å. Such channels may still be biologically active because they allow at least adaptive penetration of a water molecule (radius ~1.4 Å) upon dynamical changes. If two channels shared more than 70% of their length, only the shortest one was reported. This feature eliminated very similar (duplicate) channels. Full details of the setup of all the software tools and post-processing of results are provided in the Additional file 1. We used the same start points for all the software tools (in Additional file 1: Table S2).

**Figure 6:**
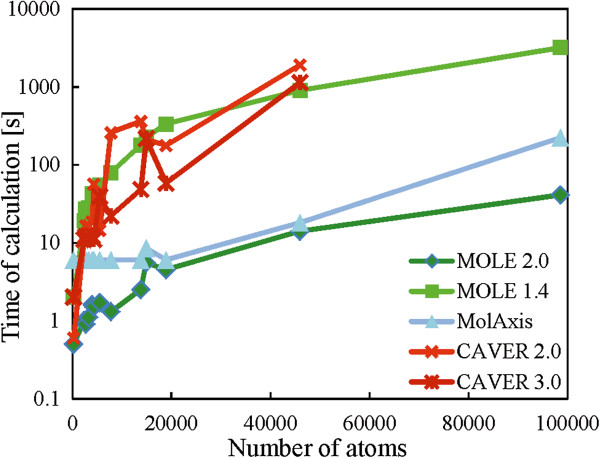
**Performance of software tools.** Time taken for the channel calculation with respect of the number of atoms in a biomacromolecule (*cf.* Additional file 1: Table S3).

Both versions of MOLE (2.0 and 1.4) together with MolAxis were able to process the largest molecular system considered in the benchmarking, i.e., the large ribosomal subunit containing almost 100,000 atoms. Consistently, MOLE 2.0 displayed the shortest processing times for both small and large systems. For small systems, MOLE 2.0 gave similar processing times to those of MolAxis (one order of magnitude faster than the CAVER tools), whereas for large systems, MOLE 2.0 was one order of magnitude faster than MolAxis and the CAVER tools were not able to calculate the largest system (large ribosomal subunit 1JJ2) (Figure 6 and Additional file 1: Table S3). Such enhancement of processing times may be a considerable advantage if a large number of structures need to be processed (e.g., in analyses of structures from molecular dynamics simulations).

MOLE 2.0 found channels in all the tested molecules, whereas the other software tools did not detect any channels in some cases: MOLE 1.4 and MolAxis in three cases, CAVER 2.0 in six cases and CAVER 3.0 in five cases (Figure 5 and Additional file 1: Table S4). All software tools predicted a rather similar set of channels. The software tools that had end points localized directly on the convex hull (e.g., MOLE 1.4, CAVER 2.0) predicted longer channels with large radii where the probe left the biomacromolecular surface (this behavior could be easily recognized from the “bulky ends” of the identified channels outside the structure). In the case of gramicidin D, which forms a transmembrane pore, MolAxis and CAVER 2.0 predicted a clearly incorrect set of channels, whereas the other tools identified appropriate channels inside the pore. It should be noted that MOLE 2.0 has a new feature of automatic identification of pores in a biomacromolecular structure, which makes it easier to characterize pores and avoids the need for manually merging two (or more) channels into a single pore (a process that cannot be overlooked if one wants to analyze pores with software tools primarily designed for the analysis of channels rather than pores).

**Figure 7:**
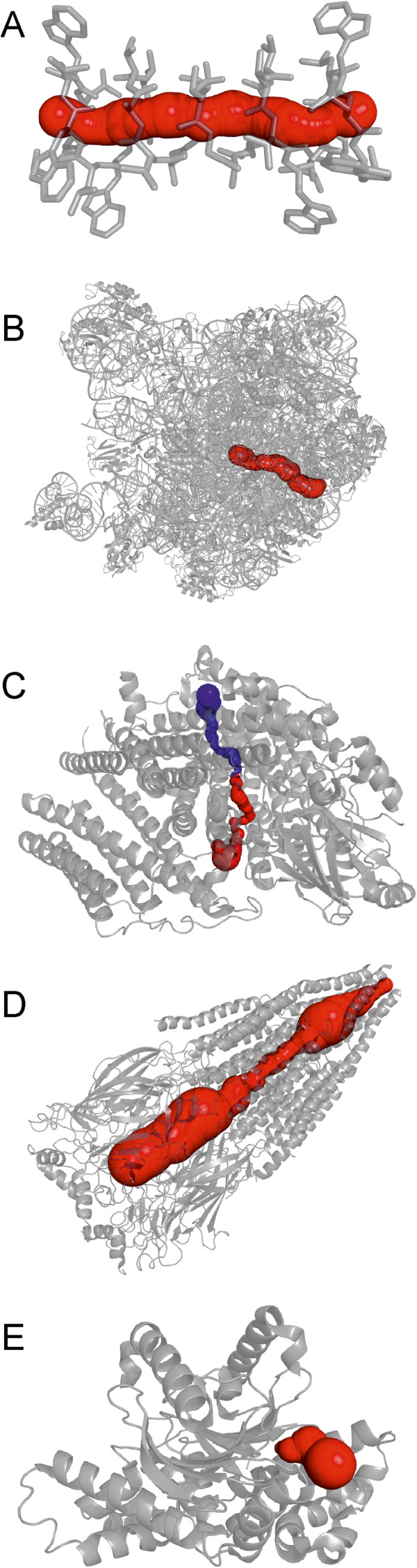
**Found channels. A**–gramicidin D (1GRM), **B**–large ribosomal subunit (1JJ2), **C**–cytochrome c oxidase (1M56), **D**–nicotinic acetylcholine receptor (2BG9), **E**–carbonic anhydrase (3EYX) by MOLE 2.0. Nonpolar channel in cytochrome c oxidase structure is shown in blue, polar channel is shown in red.

For several of the molecules containing biologically important channels/pores with known functionality and properties, we evaluated the physicochemical properties by MOLE 2.0 and related them to the known function of the channel/pore (Figure 7 and Table 2).

•Gramicidin D (1GRM) is known to form a polar pore in membranes (Figure 7A), [50] which was also reflected in the physicochemical properties identified using MOLE 2.0 as the polar part of the pore surface was predicted to be 100%. However, the predicted polarity of the pore was not high.

•The ribosomal polypeptide (1JJ2) exit channel directs a nascent protein from the proteosynthetic center to the outside of the ribosome [9]. MOLE 2.0 showed that the channel (Figure 7B) is highly polar and lined by amino acids side chains bearing positive charges (7 arginines). In addition, the channel is also lined by 16 RNA backbone phosphate groups. This clearly suggests a fragmental charge along the channels, which is necessary to prevent the nascent peptide from sticking to the channel wall inside the ribosome.

•In the cytochrome c oxidase (1M56), MOLE 2.0 identified two channels with different polarities (Figure 7C), which may be involved in the transfer process required for the proper functioning of this enzyme [51].

•The central pore (Figure 7D) of the nicotinic acetycholine receptor (2BG9) was suggested to be lined by 18 negatively charged amino acids, which explains the experimentally observed selectivity for cation permeation [52].

•The final analyzed channel was present in carbonic anhydrase (3EYX), which can utilize inorganic carbon sources CO_2_ and HCO_3_−^−^[53]. MOLE 2.0 predicted that the channel (Figure 7E) is highly polar, in agreement with expectations.

**Table 2:** Physicochemical properties of the studied biologically important channels/pores

**PDB**	**Length (Å)**	**Hydropathy**	**Hydrophobicity**	**Polarity**	**Charge**	**Mutability**	**Polar length**	**Nonpolar length**
1GRM	25.2	−0.4	−0.8	3.38	0 (0–0)	-	100%	0%
1JJ2	79.8	−1.7	−0.6	20.8	4 (6–2)^c^	68	92%	8%
1M56^a^	36.2	3.0	1.0	0.6	0	83	4%	96%
1M56^b^	41.9	1.3	0.8	12.3	0	84	48%	52%
2BG9	143.7	−1.1	−0.2	22.3	−8 (10–18)	85	81%	19%
3EYX	11.5	0.1	0.1	17.0	1 (2–1)	73	100%	0%

^a^ the nonpolar channel in Figure 7C (blue), ^b^ the polar channel in Figure 7C (red), ^c^ MOLE 2.0 counts the charge on amino acids only, whereas the ribosome channel is also lined by 16 phosphates.

Taken together, the above findings indicate that physicochemical properties may provide useful information about the nature of the channel and its biological function. However, the predicted physicochemical properties may be highly sensitive to the choice of X-ray structure, as discussed later.

### Case study: properties of channels in cytochrome P450 BM3 and P450cam

Cytochrome P450s (P450) are heme-containing monoxygenases the active sites of which are deeply buried inside their structures [11,54] and are connected to the exterior by access channels [15]. Hence, channels are considered to play an important role in the metabolism of P450 substrates [12]. Two bacterial cytochrome P450 enzymes - P450cam (CAM, which is also known as CYP101) [55] and P450 BM3 (BM3, which is also known as CYP102) [56]-have been extensively studied by X-ray diffraction in both ligand-free and ligand-bound states; to date, more than 80 structures have been published. Thus, both cytochrome P450s are suitable systems for testing the performance of MOLE 2.0 in predicting the physicochemical properties of channels.

#### Channel families

More channels were identified in BM3 than in CAM structures. As each independent chain within an asymmetric unit can have different channels [57], it is worthwhile testing all chains within a crystal structure for channel identification. Therefore, we analyzed all 80 chains within the 37 BM3 crystal structures and 54 chains within the 43 CAM crystal structures. It should be noted that CAM can be found in either closed or open states, which differ in the conformation of the F/G loop. Channels were found (using the setup described in the Methods section) only in the open CAM structures (i.e., only in 5 crystal structures: 1K2O, 1PHA, 1QMQ, 1RE9 and 1RF9).

**Figure 8:**
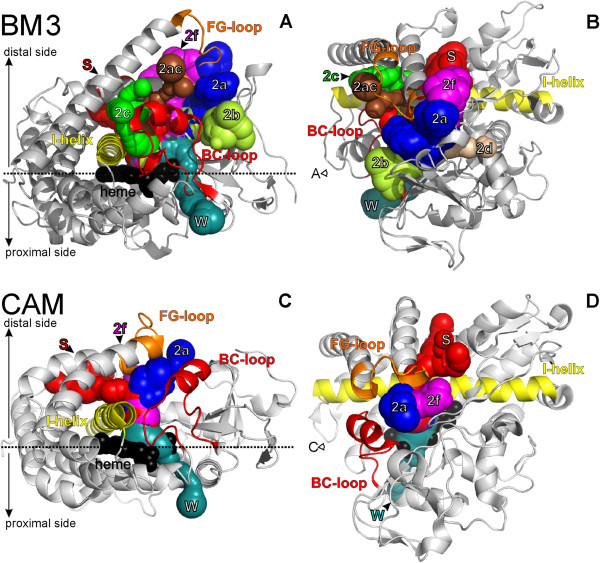
**Cytochrome P450 access and egress channels calculated by MOLE 2.0.** Channels were imposed on a cartoon representation of structures of cytochrome P450 BM3 (in views **A** and **B**; PDB structure 1BU7 was used) and cytochrome P450 CAM (in views **C** and **D**; PDB 1RE9 was used). Important secondary structures are colored as follows: the I helix is yellow, the F/G-loop is orange, the B/C-loop is red and the heme cofactor is shown as black balls. The images on the left **(A** and **C)** show views from the side in a plane horizontal to the plane of the heme; the images on the right **(B** and **D)** show views from above the distal side. Arrows indicate the viewpoints of the respective images. Channels are shown as connected spheres colored as follows: on the proximal side, channel W is colored in cyan; on the distal side channel S is shown in red; 2a–blue; 2ac–brown; 2b–light green; 2c–green; 2d–pink; 2f–magenta.

CYP structures contain several different types of active site access channels, which have been classified according to their position in relation to conserved secondary structures in the cytochrome P450 fold by Wade and coworkers [15]. There are two specifically named channels, which are considered to enable the exchange of water molecules between the active site and the enzyme exterior, i.e., the water channel neighboring the B-helix, which is the only channel leading to the CYP proximal side [12], and the solvent channel between the β4 sheet, F and I helices. Other channels are labeled by numerals and only those that are present either in CAM or BM3 structures are noted here. Channels close to the B/C and F/G loops belong to the 2× family–channel 2a is located close to the β1 sheet, F/G and B/B’ loops and it has been suggested to be the main access channel of CAM [58,59]; channel 2f neighbors channel 2a and the solvent channel and it is located between the β5 sheet and F/G loop; channel 2b also neighbors channel 2a and is located between the B/C loop, β1 and β3 sheets; channel 2c neighbors channel 2a and is located close to the B/C loop, G and I helices; channel 2ac connects channels 2a and 2c and is located between the B/C and F/G loops; channel 2d is located between the N-terminus and A helix (Figure 8).

### Variability of results

We identified 209 channels along with 73 duplicates within the 80 BM3 chains. Such a large number of channels allowed us to analyze the variability in geometrical or physicochemical properties of the identified channels between individual X-ray structures of a specific protein. The variability was evaluated as the standard deviation calculated for each channel type (W, S, 2a, 2b, 2c, 2ac, 2d, 2f). Then, the total standard deviation of a given property was calculated as a channel-number weighted average of the channels’ individual standard deviations. We also calculated the relative variability as the total standard deviation divided by the channel-number weighted mean value of a given property.

•The channel length variation was usually between 10% and 20% of the average channel length, i.e., around 5 Å in the case of BM3.

•The bottleneck radius showed a deviation of about ± 0.23 Å (less than 15%).

•The variability in the distance of bottlenecks from the start point was rather large, i.e., up to 8 Å (53%). This is not surprising because the position of bottlenecks is sensitive to the actual structure of the channel (and conformation of the lining amino acids side chains), i.e., it depends on the choice of X-ray structure [14]. The large variability in the position of bottlenecks has been also identified in molecular dynamics simulations [60]. Based on the large variability of this parameter, we do not recommend that this parameter is viewed as a robust feature of any channel found in only one crystal structure.

•The charge along a channel exhibited a deviation in the order of 0.6 *e* (about 21%).

•The hydropathy index of amino acids ranges between hydrophilic (−4.5) and hydrophobic (4.5). The variation of this value was in the order of 0.5 (less than 9%).

•The hydrophobicity index is a similar measure to the hydropathy index but has a smaller range of values between hydrophilic (−1.14) and hydrophobic amino acids (1.81). It exhibited a lower variation than the hydropathy index of about 0.14. However, its relative error was similar (less than 9%). It also seemed to be more consistent between systems as values for the same types of channels did not differ much between both proteins.

•Polarity values range from 0 for nonpolar amino acids through values of about 2 for polar amino acids towards values around 50 for charged amino acids. Polarity can therefore easily distinguish between polar channels and channels lined with charged amino acids. For instance, the solvent channel in BM3 was predicted to have a similar charge to that of channel 2f (−0.7 vs. -0.4). However, the solvent channel showed a significantly higher polarity index (9.4 vs. 2.0 for channel 2f). This indicates that the solvent channel is lined with more highly charged residues that cancel each other out, whereas channel 2f is mostly lined with nonpolar and polar residues. The variation of the polarity was in the order of ± 2.5. The relative error was about 47%. However, this value should be interpreted with care owing to the low polarity of the analyzed channels (the channel number weighted mean value was only 6.4 out of a possible range of 0–50).

•Mutability values range from the lowest mutability of 44 for Cys to a value of 177 for the most easily interchangeable Ser. The variation of mutability was in the order of ±3 and the relative error was the lowest of all the indices mentioned (less than 4%).

The results showed that the geometrical properties and physicochemical properties of the found channels typically varied by less than 20% except for the distance of bottlenecks from the starting point.

### Properties of CAM and BM3 channels

From a geometrical perspective, the most open channels were usually found within the open CAM structures, particularly 2a channels, which have a bottleneck radius larger than 2.6 Å. Channels belonging to the 2× family (mainly channels 2a, 2f, and in the case of BM3, channel 2b) were predicted to have bottleneck radii large enough to allow substrates/products to pass (> 2 Å) in both the CAM and BM3 structures, i.e., comparable or even larger than the solvent channel bottleneck radius (> 1.4 Å, radius of water molecule). The most closed channel was the water channel. However this does not necessarily mean that small molecules cannot pass through it as it might partially open to allow molecules to enter due to bottleneck fluctuations, as shown previously for the 2b channel within the structure of mammalian cytochrome P450 2A6 [14]. It is also worth noting that the solvent channel was predicted to be ~7 Å longer in CAM than in BM3, whereas other channels were typically longer in BM3. In contrast, the most open channels 2a and 2f in CAM were ~12 Å shorter than in BM3. However, this was partly because we used a probe radius of 3 Å to construct the overall shape of the protein, and therefore we only detected channels below this radius.

The water and solvent channels were clearly the most hydrophilic. The hydrophilicity also appeared to correlate with the polarity of the channels because the water and solvent channels were also predicted to be the most polar channels. The higher polarity index indicates that polar and charged amino acid residues line the solvent and water channels. On the other hand, the mutability index did not differ significantly between the individual channels. The mutability was also relatively high, which may indicate that the channels are lined with amino acids that can be relatively easily interchanged. This finding is in accord with the relatively low sequence homology between individual members of CYP family [60].

Ranking the channels according to their average hydrophobicity supported the hypothesis that the water and solvent channels are involved in water transfer into the active site [61], as the water channel was the most hydrophilic channel in both the CAM and BM3 structures, followed by the solvent channel (according to the hydropathy and hydrophobicity indices). BM3 was also predicted to contain the rather polar channel 2b. The more hydrophobic channels 2f and 2a were present in both the CAM and BM3 structures. Channels 2ac and 2d were more hydrophobic still. Finally, the most hydrophobic channel was channel 2c. However, the last three channels were found rather infrequently, i.e., only present in some BM3 structures (Additional file 1: Tables S5 and S6).

## Conclusions

We present the advanced software tool MOLE 2.0 designed to analyze molecular channels and pores. We benchmarked MOLE 2.0 against similar software tools and showed that by comparison it is faster and capable of analyzing large and complex systems containing up to hundreds of thousands of atoms. As a new feature, MOLE 2.0 estimates physicochemical properties of the identified channels. We compared the estimated physicochemical properties with the known functions of selected biomacromolecular channels and concluded that the properties correlated with the functions. We also assessed the variability of physicochemical properties by analyzing a large number of X-ray structures of two members of the cytochrome P450 superfamily. We propose that the physicochemical properties may provide useful clues about the potential functions of identified channels. The software is available free of charge at http://mole.chemi.muni.cz.

## Availability and requirements

**Project name:** MOLE 2.0

**Project home page:**http://mole.chemi.muni.cz

**Operating systems:** Mac OS, Linux, Windows

**Programming language:** C#

**Other requirements:** NET 4.0 for Windows based systems, Mono framework 2.10. or newer (http://www.mono-project.com) for other OS.

**License:** MOLE 2.0 license

**Restrictions:** free of charge

## Abbreviations

BM3: Cytochrome P450 BM3; CAM: Cytochrome P450cam; : all amino acids are represented by their respective three-letter abbreviations.

## Competing interests

The authors declare that they have no competing interests.

## Authors’ contributions

All authors contributed extensively to the work presented in this paper. DS wrote MOLE 2.0 application. All authors read and approved the final manuscript.

## Supplementary Material

Additional file 1: Table S1 Physicochemical properties of amino acids residues, setup of all software tools used for the benchmarking study. **Table S2.** Channel starting points used in the benchmarking study. **Table S3.** Duration of channel calculations for all biomacromolecules used in the benchmarking study. **Table S4.** Numbers of channels found in the analyzed molecules in the benchmarking study. **Table S5.** Comparison of geometrical and physicochemical properties of channels detected in CAM structures. **Table S6.** Comparison of geometrical and physicochemical properties of channels detected in BM3 structures. Click here for file
