# Supplementary material for: MOLE 2.0: advanced approach for analysis of biomacromolecular channels
Source: J Cheminform. 2013 Aug 16;5:39. doi: 10.1186/1758-2946-5-39 (PMC3765717; doi:10.1186/1758-2946-5-39)
Supplement: Additional file 1: Table S1 — Physicochemical properties of amino acids residues, setup of all software tools used for the benchmarking study. Table S2. Channel starting points used in the benchmarking study. Table S3. Duration of channel calculations for all biomacromolecules used in the benchmarking study. Table S4. Numbers of channels found in the analyzed molecules in the benchmarking study. Table S5. Comparison of geometrical and physicochemical properties of channels detected in CAM structures. Table S6. Comparison of geometrical and physicochemical properties of channels detected in BM3 structures. [file 1758-2946-5-39-S1.docx]

Additional file

MOLE 2.0: Advanced Approach for Analysis of Biomacromolecular Channels

David Sehnal†,$, Radka Svobodová Vařeková†, Karel Berka%, Lukáš Pravda†, Veronika Navrátilová%, Pavel Banáš%, Crina-Maria Ionescu†, Stanislav Geidl†, Michal Otyepka%,*, Jaroslav Koča†,*

†National Centre for Biomolecular Research, Faculty of Science and CEITEC - Central European Institute of Technology, Masaryk University Brno, Kamenice 5, 625 00 Brno-Bohunice, Czech Republic

$ Faculty of Informatics, Masaryk University Brno, Botanická 68a, 602 00 Brno, Czech Republic

% Regional Centre of Advanced Technologies and Materials, Department of Physical

Chemistry, Faculty of Science, Palacký University Olomouc, tř. 17. listopadu 12, 771 46,

Olomouc, Czech Republic.

* Corresponding authors:

MO: Phone: +420 585634756, fax: +420 585634761, e-mail: [michal.otyepka@upol.cz](mailto:michal.otyepka@upol.cz);

JK: Phone: +420 549492685, fax: +420 549491060, e-mail: [jkoca@chemi.muni.cz](mailto:jkoca@chemi.muni.cz)

**Benchmarking Study Setup**

**MOLE 2.0** was executed with *Origin Radius* 5 Å, *Interior Threshold* 1.25 Å, *Surface Cover Radius* 10 Å, *Bottleneck Tolerance* 3 Å, *Bottleneck Radius* 1.25 Å, *Cutoff Ratio* 0.7 Å and *Probe Radius* 3 Å. For larger biomacromolecules, higher values of *Probe Radius* were used as these molecules can contain wide channel. *Probe Radius* 5 Å was used for 1JJ2, 1S5L; 6 Å was used for 2CQT; and 8 Å was used for 1SU4 and 2BG9.

**MOLE 1.4** and **CAVER 2.0** do not allow additional tuning of the calculation. We kept the default values with exception to *Origin Radius* set to 5 Å. Moreover, these tools require a priori knowledge of the number of channels to be returned. For this reason, we have select calculation of 20 channels.

**MolAxis** introduces more parameters than two previously mentioned tools; most of them were set as default with exception to *Origin Radius* (5 Å) and *Probe Radius* (1.25 Å; this parameter represents similar behavior as *Bottleneck Radius* in the case of MOLE 2.0). Next exception is *Bounding Sphere Radius*, which was adjusted to 70 Å for larger structures (1JJ2, 2BG9) and which remain default (30 Å) for the rest of structures.

**CAVER 3.0** introduces the largest number of parameters to set. CAVER 3.0 filtering according to the radius of the channel is extremely strict. Therefore, *Probe Radius* 0.9 Å (similar to *Bottleneck radius* in the case of MOLE 2.0) was used instead of 1.25 Å, which allows to find more channels. The *Origin Radius* was set the same as in previous cases (5 Å). Additional startup parameters were adjusted for large molecules (see below; 1JJ2, 1S5L, 2CQT, 1SU4, 2BG9) in order to determine reasonably the best results in a reasonable time.

| structure | 1JJ2 | 1S5L | 2CQT | 1SU4 | 2BG9 |
| --- | --- | --- | --- | --- | --- |
| clustering_threshold | 8.5 | 8.5 | 3.5 | 3.5 | 3.5 |
| shell_depth | 4.0 | 4.0 | 2.0 | 2.0 | 4.0 |
| shell_radius | 15.0 | 15 | 3.0 | 3.0 | 15.0 |
| number_of_approximating_balls | 4 | 4 | 4 | 4 | 4 |

The results of all the tools with exception of MOLE 2.0 were further processed in order to get the same coverage of centerline as with MOLE 2.0 by natural cubic spline.

Furthermore the results of all the tools were processed by additional filter in order to filter out identical channels and also those which do not meet allowed length of 3 Å of the bottleneck bellow 1.25 Å (similarly as with MOLE 2.0 parameters *Bottleneck Radius* 1.25 Å and *Bottleneck Length* 3.0 Å). Finally, in the case that the channels shared more than 70% identity in their length, the longer one was discarded.

All channels were calculated on a computer with a processor Intel Core i5 430M-2.26GHz and 4 GB RAM.

**Table S1.** Physicochemical properties amino acids taken from literature.

| Amino acid | | | Charge | Hydropathy^a^ | Hydrophobicity^b^ | Polarity^c^ | Mutability^d^ |
| --- | --- | --- | --- | --- | --- | --- | --- |
| Side chains | ALA | A | 0 | 1.8 | 0.02 | 0 | 100 |
|  | ARG | **R** | **+1** | −4.5 | -0.42 | 52.0 | 83 |
|  | ASN | N | 0 | −3.5 | -0.77 | 3.38 | 104 |
|  | ASP | **D** | **−1** | −3.5 | −1.04 | 49.7 | 86 |
|  | CYS | C | 0 | 2.5 | 0.77 | 1.48 | 44 |
|  | GLU | **E** | **−1** | −3.5 | −1.14 | 49.9 | 77 |
|  | GLN | Q | 0 | −3.5 | −1.10 | 3.53 | 84 |
|  | GLY | G | 0 | −0.4 | −0.80 | 0 | 50 |
|  | HIS | H | 0 | −3.2 | 0.26 | 51.6 | 91 |
|  | ILE | I | 0 | 4.5 | 1.81 | 0.13 | 103 |
|  | LEU | L | 0 | 3.8 | 1.14 | 0.13 | 54 |
|  | LYS | **K** | **+1** | −3.9 | −0.41 | 49.5 | 72 |
|  | MET | M | 0 | 1.9 | 1.00 | 1.43 | 93 |
|  | PHE | F | 0 | 2.8 | 1.35 | 0.35 | 51 |
|  | PRO | P | 0 | −1.6 | −0.09 | 1.58 | 58 |
|  | SER | S | 0 | −0.8 | −0.97 | 1.67 | 117 |
|  | THR | T | 0 | −0.7 | −0.77 | 1.66 | 107 |
|  | TRP | W | 0 | −0.9 | 1.71 | 2.10 | 25 |
|  | TYR | Y | 0 | −1.3 | 1.11 | 1.61 | 50 |
|  | VAL | V | 0 | 4.2 | 1.13 | 0.13 | 98 |
| Main chain | | | 0 | −0.4 | −0.80 | 3.38 | − |

a) Kyte, J.; Doolittle, R. F. J. Mol. Biol. 1982, 157, 105–132.

b) Cid, H.; Bunster, M.; Canales, M.; Gazitúa, F. Protein Eng. 1992, 5, 373–375.

c) Jones, D. T.; Taylor, W. R.; Thornton, J. M. Comput. Appl. Biosci. 1992, 8, 275–82.

d) Zimmerman, J. M.; Eliezer, N.; Simha, R. J. Theor. Biol. 1968, 21, 170–201.

**Table S2.** Starting point definitions used in benchmark comparison between all software tools.

| PDB ID | Starting point |
| --- | --- |
| 1TQN | A (GLU 308, THR 309) |
| 1M56 | G (HIS 284, TYR 288, HIS 419, PHE 420, HIS 421, ARG 481, ARG 482) |
| 2CQT | B GLU 503 |
| 2ACE | A (SER 200, GLU 327, HIS 440) |
| 1CQW | A (ASP 117, TRP 118, GLU 141, HIS 283) |
| 1SU4 | A ASP 351 |
| 3EYX | A (ASP 59, ARG 61) |
| 1GRM | [-0.018, -0.012, 4.197] |
| 2BG9 | [63.42, 63.73, 111.83] |
| 3CAP | [14.383, -32.452, -32.102] |
| 2OJ3 | [29.914, 46.439, 84.401] |
| 1JJ2 | [69.84, 131.02, 84.46] |
| 1S5l | [31.296, 43.090, 65.308] |

**Table S3.** Time of channel calculations in tested biomacromolecules.

| PDBID | Number of Atoms | Time of calculation [s] | | | | |
| --- | --- | --- | --- | --- | --- | --- |
|  |  | MOLE 2.0 | MOLE 1.4 | MolAxis | CAVER 2.0 | CAVER 3.0 |
| 1GRM | 272 | 0.5 | 2 | 6 | 0.6 | 2 |
| 2OJ3 | 2386 | 1 | 19 | 6 | 11.8 | 11 |
| 1CQW | 2754 | 0.9 | 27 | 6 | 16.5 | 12 |
| 3EYX | 3308 | 1.1 | 28 | 6 | 16.5 | 12 |
| 1TQN | 3999 | 1.6 | 42 | 6 | 18.5 | 12 |
| 2ACE | 4357 | 1.5 | 42 | 6 | 56.5 | 11 |
| 3CAP | 5504 | 1.7 | 55 | 6 | 14.5 | 39 |
| 1SU4 | 7791 | 1.3 | 79 | 6 | 259 | 22 |
| 2CQT | 13833 | 2.5 | 179 | 6 | 360 | 48 |
| 2BG9 | 14924 | 5.5 | 225 | 8.5 | 212 | 215 |
| 1M56 | 18934 | 4.5 | 333 | 6 | 177 | 58 |
| 1S5L | 45945 | 14 | 897 | 18 | 1902 | 1127 |
| 1JJ2 | 98543 | 41 | 3180 | 222 | - | - |

**Table S4.** Number of channels found in the analyzed molecules.

| PDB ID | Number of channels found | | | | |
| --- | --- | --- | --- | --- | --- |
|  | MOLE 2.0 | MOLE 1.4 | MolAxis | CAVER2.0 | CAVER 3.0 |
| 1TQN | 3 | 3 | 3 | 4 | 1 |
| 1M56 | 1 | 0 | 5 | 5 | 0 |
| 2CQT | 2 | 2 | 1 | 0 | 2 |
| 2ACE | 1 | 0 | 0 | 0 | 0 |
| 1CQW | 2 | 0 | 1 | 0 | 0 |
| 1SU4 | 2 | 6 | 2 | 0 | 1 |
| 3EYX | 1 | 2 | 2 | 0 | 2 |
| 1GRM | 2 | 2 | 4 | 1 | 2 |
| 2BG9 | 9 | 6 | 1 | 4 | 17 |
| 3CAP | 3 | 7 | 0 | 4 | 3 |
| 2OJ3 | 2 | 3 | 0 | 1 | 0 |
| 1JJ2 | 17 | 5 | 12 | 0 | 0 |
| 1S5L | 3 | 2 | 2 | 1 | 2 |

**Table S5.** Comparison of geometrical and physicochemical properties of channels detected in CAM structures.

| CAM Channel | S | | | W | | | 2a | | | 2f | | |
| --- | --- | --- | --- | --- | --- | --- | --- | --- | --- | --- | --- | --- |
| Number + Duplicates | 4 | + | 3 | 3 | + | 0 | 4 | + | 0 | 2 | + | 0 |
| Length [Å] | 27.2 | ± | 1.7 | 25.1 | ± | 1.6 | 17.2 | ± | 4.2 | 19.1 | ± | 3.2 |
| Min – Max | 24.8 | – | 28.9 | 23.5 | – | 26.7 | 13.5 | – | 23.1 | 16.8 | – | 21.4 |
| Bottleneck R | 1.50 | ± | 0.38 | 1.16 | ± | 0.09 | 2.24 | ± | 0.55 | 2.17 | ± | 0.15 |
| Min – Max [Å] | 1.16 | – | 1.94 | 1.06 | – | 1.24 | 1.43 | – | 2.63 | 2.06 | – | 2.27 |
| Charge | -1.5 | ± | 0.6 | 0.7 | ± | 0.6 | -0.3 | ± | 0.5 | 1.0 | ± | 0.0 |
| Min – Max | -2 | – | -1 | 0 | – | 1 | -1 | – | 0 | 1 | – | 1 |
| Hydropathy | 0.07 | ± | 0.58 | -0.72 | ± | 0.35 | 1.88 | ± | 0.26 | 1.20 | ± | 0.36 |
| Min – Max | -0.45 | – | 0.90 | -1.00 | – | -0.33 | 1.62 | – | 2.19 | 0.94 | – | 1.45 |
| Hydrophobicity | -0.02 | ± | 0.16 | -0.25 | ± | 0.08 | 0.67 | ± | 0.11 | 0.31 | ± | 0.07 |
| Min – Max | -0.14 | – | 0.22 | -0.32 | – | -0.16 | 0.52 | – | 0.77 | 0.26 | – | 0.36 |
| Polarity | 17.8 | ± | 3.8 | 14.1 | ± | 2.1 | 1.7 | ± | 1.4 | 4.8 | ± | 3.3 |
| Min – Max | 12.6 | – | 21.8 | 11.8 | – | 16.0 | 0.9 | – | 3.7 | 2.5 | – | 7.1 |
| Mutability | 83.8 | ± | 1.0 | 83.7 | ± | 1.5 | 78.3 | ± | 2.2 | 82.5 | ± | 3.5 |
| Min – Max | 83 | – | 85 | 82 | – | 85 | 76 | – | 81 | 80 | – | 85 |
| Bottleneck D | 21.1 | ± | 5.2 | 15.7 | ± | 1.5 | 15.2 | ± | 3.4 | 18.5 | ± | 3.8 |
| Min – Max [Å] | 13.4 | – | 24.0 | 14.1 | – | 17.1 | 12.1 | – | 19.9 | 15.9 | – | 21.2 |

Geometrical properties include (i) number of detected channels of each type, (ii) number of duplicate channels, (iii) length of the channel, (iv) bottleneck radius (bottleneck R), and (v) the distance of the bottleneck (bottleneck D) from the starting point (heme) along the channel path. Physicochemical properties include (i) sum of charges of unique amino acids lining the channel, (ii) average hydropathy, (iii) average hydrophobicity, (iv) average polarity, and (v) average mutability indices calculated for unique amino acid residues lining the channel path. The nomenclature of channels by Wade et al. [Cojocaru V, Winn PJ, Wade RC: The ins and outs of cytochrome P450s. *Biochimica et Biophysica Acta* 2007, 1770:390–401] is used. Channels are colored as follows: channel S is shown in red; W - cyan; 2a – blue; 2f – magenta.

**Table S6.** Comparison of geometrical and physicochemical properties of channels detected in BM3 structures.

| Property | S | | | | W | | | 2a | | | 2f | | | 2b | | | 2d | | | 2ac | | | 2c | | |
| --- | --- | --- | --- | --- | --- | --- | --- | --- | --- | --- | --- | --- | --- | --- | --- | --- | --- | --- | --- | --- | --- | --- | --- | --- | --- |
| Number + Duplicates | 32 | + | 6 | 38 | | + | 1 | 27 | + | 6 | 41 | + | 6 | 63 | + | 3 | 3 | + | 4 | 2 | + | 0 | 3 | + | 0 |
| Length [Å] | 19.7 | ± | 3.5 | 31.4 | | ± | 2.8 | 30.2 | ± | 4.8 | 28.7 | ± | 4.4 | 23.8 | ± | 8.4 | 29.6 | ± | 3.5 | 30.8 | ± | 5.8 | 25.1 | ± | 3.2 |
| Min – Max | 11.6 | – | 27.0 | 26.9 | | – | 38.0 | 14.9 | – | 36.9 | 17.7 | – | 37.2 | 7.2 | – | 34.2 | 25.8 | – | 32.5 | 26.7 | – | 34.9 | 22.6 | – | 28.7 |
| Bottleneck R | 1.42 | ± | 0.19 | 1.17 | | ± | 0.04 | 1.59 | ± | 0.37 | 1.67 | ± | 0.31 | 1.72 | ± | 0.26 | 1.55 | ± | 0.05 | 1.13 | ± | 0.12 | 1.30 | ± | 0.03 |
| Min – Max [Å] | 1.07 | – | 1.77 | 1.11 | | – | 1.29 | 1.04 | – | 2.39 | 1.04 | – | 2.39 | 1.18 | – | 2.55 | 1.50 | – | 1.60 | 1.04 | – | 1.21 | 1.27 | – | 1.33 |
| Charge | -0.7 | ± | 0.7 | 0.5 | | ± | 0.5 | 0.9 | ± | 0.5 | -0.4 | ± | 0.7 | 0.4 | ± | 0.6 | 0.0 | ± | 1.0 | 0.5 | ± | 0.7 | -0.3 | ± | 1.2 |
| Min – Max | -2 | – | 1 | 0 | | – | 1 | -1 | – | 2 | -2 | – | 1 | -1 | – | 2 | -1 | – | 1 | 0 | – | 1 | -1 | – | 1 |
| Hydropathy | 0.59 | ± | 0.44 | -0.21 | | ± | 0.36 | 1.11 | ± | 0.33 | 1.04 | ± | 0.41 | 0.79 | ± | 0.65 | 1.94 | ± | 0.10 | 1.78 | ± | 0.10 | 1.83 | ± | 0.71 |
| Min – Max | -0.15 | – | 1.63 | -1.08 | | – | 0.43 | 0.07 | – | 1.70 | -0.08 | – | 1.72 | -0.37 | – | 2.13 | 1.83 | – | 2.03 | 1.71 | – | 1.85 | 1.31 | – | 2.64 |
| Hydrophobicity | 0.00 | ± | 0.19 | -0.22 | | ± | 0.09 | 0.29 | ± | 0.11 | 0.19 | ± | 0.15 | 0.13 | ± | 0.14 | 0.59 | ± | 0.04 | 0.55 | ± | 0.02 | 0.60 | ± | 0.30 |
| Min – Max | -0.26 | – | 0.50 | -0.44 | | – | -0.06 | -0.01 | – | 0.54 | -0.11 | – | 0.45 | -0.20 | – | 0.40 | 0.56 | – | 0.63 | 0.53 | – | 0.56 | 0.35 | – | 0.93 |
| Polarity | 9.4 | ± | 3.2 | 12.8 | | ± | 2.7 | 3.8 | ± | 1.6 | 2.0 | ± | 1.2 | 5.2 | ± | 3.4 | 2.2 | ± | 1.3 | 4.5 | ± | 1.3 | 3.8 | ± | 2.7 |
| Min – Max | 4.2 | – | 18.0 | 8.6 | | – | 19.2 | 1.2 | – | 8.6 | 0.9 | – | 5.6 | 0.7 | – | 12.2 | 0.8 | – | 3.4 | 3.6 | – | 5.4 | 1.1 | – | 6.5 |
| Mutability | 84.9 | ± | 2.7 | 81.5 | | ± | 2.1 | 78.7 | ± | 3.3 | 81.5 | ± | 3.4 | 83.9 | ± | 3.3 | 75.3 | ± | 0.6 | 73.5 | ± | 2.1 | 84.7 | ± | 7.2 |
| Min – Max | 81 | – | 93 | 76 | | – | 86 | 73 | – | 86 | 77 | – | 88 | 71 | – | 92 | 75 | – | 76 | 72 | – | 75 | 80 | – | 93 |
| Bottleneck D | 11.6 | ± | 2.9 | 18.3 | | ± | 3.3 | 18.9 | ± | 11.8 | 14.8 | ± | 10.6 | 14.4 | ± | 11.2 | 27.5 | ± | 3.7 | 21.7 | ± | 4.1 | 18.9 | ± | 2.5 |
| Min – Max [Å] | 6.2 | – | 17.1 | 13.7 | | – | 32.7 | 3.0 | – | 33.0 | 1.6 | – | 30.4 | 1.8 | – | 30.6 | 23.5 | – | 30.7 | 18.8 | – | 24.6 | 17.0 | – | 21.8 |

The geometrical and physicochemical properties studied are as described in Table 4. The nomenclature of channels by Wade et al. [Cojocaru V, Winn PJ, Wade RC: The ins and outs of cytochrome P450s. *Biochimica et Biophysica Acta* 2007, 1770:390–401] is used. Channels are colored as follows: channel S is shown in red; W - cyan; 2a – blue; 2f – magenta; 2b – light green; 2d – pink; 2ac – brown; 2c – green.
